# Sample Entropy Analysis of Noisy Atrial Electrograms during Atrial Fibrillation

**DOI:** 10.1155/2018/1874651

**Published:** 2018-06-13

**Authors:** Eva María Cirugeda-Roldán, Antonio Molina Picó, Daniel Novák, David Cuesta-Frau, Vaclav Kremen

**Affiliations:** ^1^Technological Institute of Informatics, Universitat Politècnica de València, Alcoi Campus, Plaza Ferrándiz y Carbonell 2, Alcoi, Spain; ^2^Department of Cybernetics, Faculty of Electrical Engineering, Czech Technical University in Prague, Czech Republic; ^3^Czech Institute of Informatics, Robotics and Cybernetics, Czech Technical University in Prague, Czech Republic

## Abstract

Most cardiac arrhythmias can be classified as atrial flutter, focal atrial tachycardia, or atrial fibrillation. They have been usually treated using drugs, but catheter ablation has proven more effective. This is an invasive method devised to destroy the heart tissue that disturbs correct heart rhythm. In order to accurately localise the focus of this disturbance, the acquisition and processing of atrial electrograms form the usual mapping technique. They can be single potentials, double potentials, or complex fractionated atrial electrogram (CFAE) potentials, and last ones are the most effective targets for ablation. The electrophysiological substrate is then localised by a suitable signal processing method. Sample Entropy is a statistic scarcely applied to electrograms but can arguably become a powerful tool to analyse these time series, supported by its results in other similar biomedical applications. However, the lack of an analysis of its dependence on the perturbations usually found in electrogram data, such as missing samples or spikes, is even more marked. This paper applied SampEn to the segmentation between non-CFAE and CFAE records and assessed its class segmentation power loss at different levels of these perturbations. The results confirmed that SampEn was able to significantly distinguish between non-CFAE and CFAE records, even under very unfavourable conditions, such as 50% of missing data or 10% of spikes.

## 1. Introduction

Arrhythmia is an abnormal too fast, too slow, or irregular pattern heart rate. Most cardiac arrhythmias can be classified as atrial flutter, focal atrial tachycardia, or atrial fibrillation (AF) [1], the most prevalent arrhythmia. Causes of arrhythmia vary and are diverse: coronary heart disease, smoking, diabetes, obesity, age, some medications, hypertension, etc. They have been usually treated using drugs, but catheter ablation has proven more effective, especially in patients with persistent arrhythmia. This is an invasive method devised to cauterise the heart tissue that disturbs correct heart rhythm [2].

Radiofrequency or laser catheters have to be accurately guided by 3D anatomical navigation systems to this substrate. The acquisition and processing of atrial electrograms (AEGM) form the usual mapping technique [3], with a vast disparity of models and algorithms used in practice. Specifically, the assessment of AEGM complexity plays an increasingly important role in research as it can help physicians to minimise the inconvenience of Radiofrequency Ablation (RFA) procedures. Mapping complex fractionated AEGM (CFAE) as target sites for AF ablation is promising. CFAE areas represent critical sites for AF perpetuation and can serve as target sites for AF ablation [3].

The Dominant Frequency (DF) of AEGM signals is one of the most widely used common tools in this context. Algorithms to extract DF for AF ablation have been described in [4, 5]. A new strategy has also been reprogrammed and implemented in [6]. This strategy uses the complexity evaluation of CFAE, which was first introduced in [7] plus the semiautomatic implementation of the CARTO® (Biosense Webster, Diamond Bar, CA, US) CFAE algorithm [6]. The CARTO-XP® mapping system [8] has also been reimplemented in [6]. Two separate AEGM complexity measures have been extracted, the ICL (Interval Confidence Level) and SCI (Shortest Complex Interval) indices [9]. Both indices have also been described in [7] and used in [6, 8]. A measurement of intervals between the discrete peaks of AEGM signals has also been described. These methods contribute valuable information about the level of AEGM complexity which is extracted from CFAE by the unsupervised method [6], but it is still necessary to improve the level of the autonomous classification of AEGM complexity to further help the RFA of AF navigation procedures.

Since it is a highly invasive and complex technique, AEGM signal recording can be affected by many artifacts in the acquisition stage. For example, sensor failure or movement can introduce spikes during signal recording [10, 11], where spikes are sharp impulses of linearly rising and falling edges. Given the way experts classify CFAE signals, these artifacts can bias their interpretation by assigning CFAE records to an incorrect fractionation level. Although many signal processing techniques are available to reduce artifacts such as spikes [12], sometimes this is not possible because of their striking similarity to signal features [13], and the original signal cannot be completely reconstructed [14]. The influence of spikes on complexity measures has been previously characterised for electrocardiograph and electroencephalograph records [11, 15]. In [10], a comparative study of ApEn and SampEn robustness to spikes was carried out in stochastic processes and with simulated and real RR and ECG signals.

AEGM are also prone to having gaps in their time series. Unstable positioning, poor contact, or other problems related to catheters may lead to incomplete or incorrect data [16]. Previous studies have considered random and uniform sample loss in biomedical records and can be found in [17, 18]. These studies have assessed the influence of missing data on the complexity of electroencephalograph signals. In [19], a brief study about infant heart rate signals with random sample loss is presented. Similarly, in [20], Heart Rate Variability (HRV) signals have been considered but applied a uniform sample loss to beat to beat intervals (R-R intervals) from which HRV records were extracted. No study has analysed the influence of sample loss, or spikes, on AEGM records.

This work addresses the study of the influence of possible artifacts on the separability of AEGM records using entropy estimators. The metric SampEn [21] has proven successful in this task [22] using signals from different databases, but without the artifacts stated above. In this case, we included quantitative characterisation against spikes and sample loss to assess SampEn robustness against possible unfavourable real conditions for AEGM time series. Significant performance degradation would render SampEn unusable despite the good results obtained in [22]. SampEn performance and robustness have been evaluated in statistical test and correlation coefficient terms.

The remaining sections of the paper are arranged as follows: the next Section 2 describes the SampEn algorithm in detail, the experimental dataset, the synthetic artifacts to be included in the time series, and the employed statistical assessment.  Section 3 presents the study results graphically and numerically. Discussion of these results takes place in Section 4. Finally, conclusions about the influence of perturbations on AEGM records in SampEn are drawn in Section 5.

## 2. Materials and Methods

### 2.1. Entropy Metrics

SampEn was first proposed by Richman et al. in [21]. It was devised as a solution to reduce the bias in ApEn and to, therefore, yield a more robust statistic. This new approach was based on avoiding template self-matches computing.

SampEn estimates the regularity of a time series by computing the negative logarithm of the conditional probability that two sequences, which are similar (template match[21]) for *m* points, remain similar for *m* + 1 points at a dissimilarity level under a certain threshold *r*[19, 21]. It is largely independent of record length and exhibits relative consistency in circumstances in which ApEn does not. SampEn agrees much better than ApEn statistics with the theory for random numbers over wide-ranging operating conditions [21].

Given an input time series **x** = {*x*_1_, *x*_2_,…, *x*_*N*_} of size *N*, sequences to compare are obtained by splitting **x** into epochs of length *m*, **x**_*i*_ = {*x*_*i*_, *x*_*i*+1_,…, *x*_*i*+*m*−1_}, *i* = 1,…, *N* − *m* + 1. The dissimilarity measure between two of these sequences is defined as *d*_*ij*_ = max⁡(|*x*_*i*+*k*_ − *x*_*j*+*k*_|),  0 ≤ *k* ≤ *m* − 1,  *j* ≠ *i*. Two additional parameters are required to compute SampEn: the number of matches (number of sequences *x*_*j*_ so that *d*_*ij*_ ≤ *r*) for sequences of length *m*, *B*_*i*_(*r*), and the number of matches for sequences of length *m* ← *m* + 1, *A*_*i*_(*r*). These parameters can then be averaged as

(1) Bimr=1N−m−1BirAimr=1N−m−1Air

and expressed as probabilities:

(2) Bmr=1N−m∑i=1N−mBimrAmr=1N−m∑i=1N−mAimr

SampEn can then be computed as the natural logarithm of the likelihood ratio:

(3) $$\begin{array}{c} SampEn\left(\;m,r\;\right)=\underset{N\to \infty }{\lim}\left(\;-\ln\left[\;\frac{A^{m}\left(r\right)}{B^{m}\left(r\right)}\;\right]\;\right) \end{array}$$

or for finite time series:

(4) $$\begin{array}{c} SampEn\left(\;m,r,N\;\right)=-ln\left[\;\frac{A^{m}\left(r\right)}{B^{m}\left(r\right)}\;\right] \end{array}$$

The number of matches can be increased by decreasing *m* or increasing *r*, but it may impact the ability of SampEn to discern between classes [22]. Both parameters represent a trade-off criterion between accuracy and discrimination capability, and there are no guidelines to optimally choose them. In this case, and according to [21], *m* was set to 2 and *r* = 0.2.

### 2.2. Experimental Dataset

A final database containing 113 AEGM records from 12 different patients, nine of whom were males, was used in the experiments. AEGM were preselected by an expert from a larger database recorded in a single study in the Czech Republic [6, 22], after ruling out any noisy, unstable, or artifacted records. The selection criteria were as follows:

- Good endocardial contact.
- Not close to the mitral annulus to avoid possible interferences from ventricular signals.
- No visually apparent redundancies.
- Featuring all forms: very organised, very fractionated, or intermediate.

AEGM signals were acquired in the AF mapping procedures performed on the patients indicated for RFA of AF [23]. Signals were sampled at 977 Hz and recorded by CardioLab 7000, Prucka Inc., and then resampled to 1 KHz. Each preselected AEGM signal in this dataset was 1,500 ms long. It would have been preferable to have longer records, but the expert signal selection was driven by the aim to achieve good stability and a high signal-to-noise ratio for later AEGM fractionation degree assessment by an expert. Relatively short records are a limitation of this study, but they guarantee more stability. Data were preprocessed for baseline wander and high frequency noise removal purposes.

**Figure 1:**
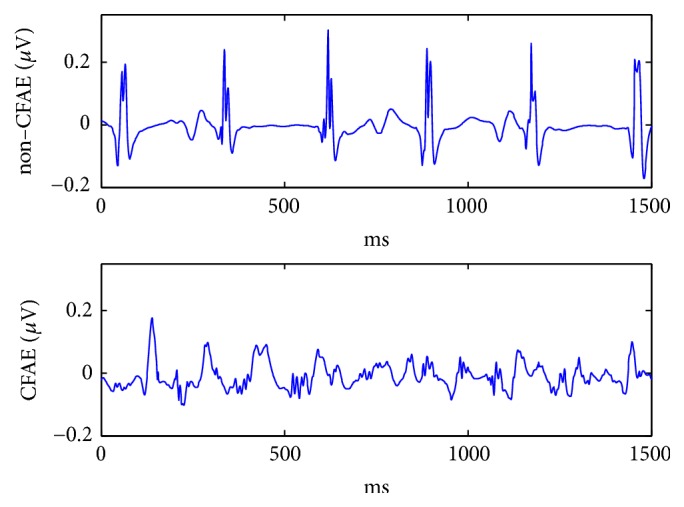
AEGM signals of each group of the database: noncomplex fractionated atrial electrogram (NC AEGM) and complex fractionated atrial electrogram (C AEGM).

According to [7, 24], AEGM were classified into two main classes: non-CFAE (NC) and CFAE (C). The first class, NC, included the AEGM recorded in regions where three independent experts (who perform AF ablation on a regular basis) would not recommend an ablative procedure to be performed (64 records, organised activity, or mild degree of fractionation). The C class contained the signals recorded in the areas where experts would ablate (49 records, intermediate or high degree of fractionation). The final classification corresponded to the average of the three experts' rankings [6]. This classification was based on the subjective perception of signals by the three experts, helped by a specific software tool that displays the AEGM grouped according to their aspect ratio [6].  Figure 1 shows a representative signal of each class considered in the database.

### 2.3. Synthetic Artifacts

#### 2.3.1. Spikes

Spikes are considered nonstationarities which may arise from external conditions that have little to do with the intrinsic dynamics of the system [10], this being the fundamental basis of the spike generation algorithm.

The presence of a spike in a train is defined by a binomial random process: *β*(*N*, *p*_*s*_), where *p*_*s*_ is the probability of a spike occurring in a time series of length *N*. Spike amplitude was defined as a uniform random variable *Ω*(−3*λ*, 3*λ*), where *λ* accounts for the peak-to-peak amplitude of the original AEGM signal. All the spikes were considered to have a fixed length of one sample [15].

**Figure 2:**
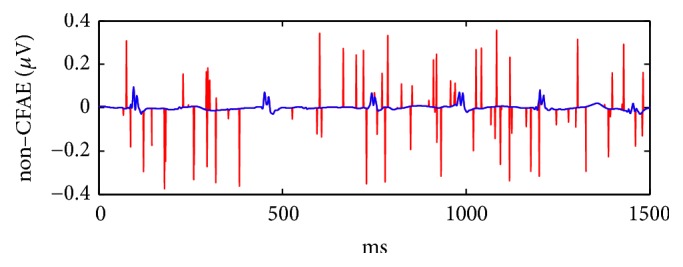
An NC signal in blue with a superimposed spike train (*p*_*s*_ = 0.05) in red.

Mathematically speaking, spike train *s*(*t*) is defined as

(5) $$\begin{array}{c} s\left(\;t\;\right)=\underset{i}{\sum }a_{i}\delta \left(\;t-t_{i}\;\right) \end{array}$$

where *a*_*i*_ is the spike amplitude obtained from *Ω* and *t*_*i*_ is the spike temporal location, generated by means of *β*. Fifty realisations of independent random spike trains were added to the AEGM original signals in the experimental data set, with probabilities *p*_*s*_ = [0.01,0.02,0.03,0.04,0.05,0.10,0.15,0.20,0.30,0.40,0.50]. For illustrative purposes, Figure 2 shows one of these realisations, where a spike train was superimposed to an NC record.

#### 2.3.2. Sample Loss

Two algorithms to generate sample losses were considered according to the realistic situations that can take place during catheter recording of the AEGM time series: distributed and consecutive sample losses. Once again, 50 realisations per signal were considered to preserve statistical properties. In both experiments, the number of samples to be removed from each signal was set at a percentage *η* of total signal length *N*. Given the similarity to the previous spike experiment, the same percentages were considered, *η*(%) = [1,2, 3,4, 5,10,15,20,30,40,50]. Due to this sample removal process, records were shortened by *ηN* samples. More specifically,

- distributed random sample loss was based on removing the isolated samples at the random locations given by *β*, until the total number of samples to be removed *ηN* was achieved.
- consecutive random sample loss was based on removing a segment of *ηN* consecutive samples. Randomness was introduced into the initial sample that was removed. This sample was selected according to *β* to ensure that it would be different in all 50 realisations of each experiment.

### 2.4. Statistical Assessment

The segmentation results were assessed using a Mann–Whitney U test [25]. Specifically, this test was used to quantify the probability of the two groups, C and NC, having the same median value. The significance threshold was set at *α* = 0.01. There was no need to check the normality of the results with this test. The performance deviation from the baseline case of no artifacts was quantified using a correlation coefficient *ρ*_*xy*_.

## 3. Results

### 3.1. No Artifacts

**Figure 3:**
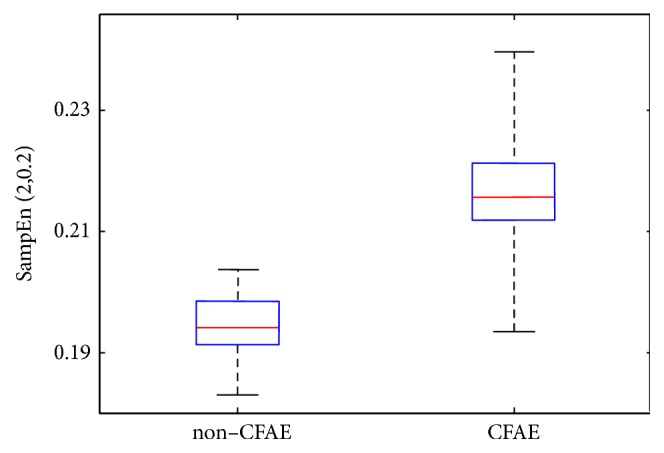
Boxplot distribution of C and NC AEGM SampEn values, with no artifact added to the experimental dataset.

By taking the value of SampEn(2,0.2) as the distinguishing characteristic, it was possible to segment between the C and NC AEGM records significantly, with a *p*-value< 0.001. As shown in Figure 3, the interquartile ranges (featured by the blue box) do not overlap and the median values (red line) are far enough to be statistically different, according to the Mann–Whitney U test results.

Even though distributions were not statistically normal, the 95% confidence intervals given by [*μ* ± 2*σ*], where *μ* is SampEn mean and *σ* is its standard deviation, do not overlap: [0.193,0.199] for neither NC nor [0.216,0.223] C AEGM.

### 3.2. Spikes Influence on SampEn

**Figure 4:**
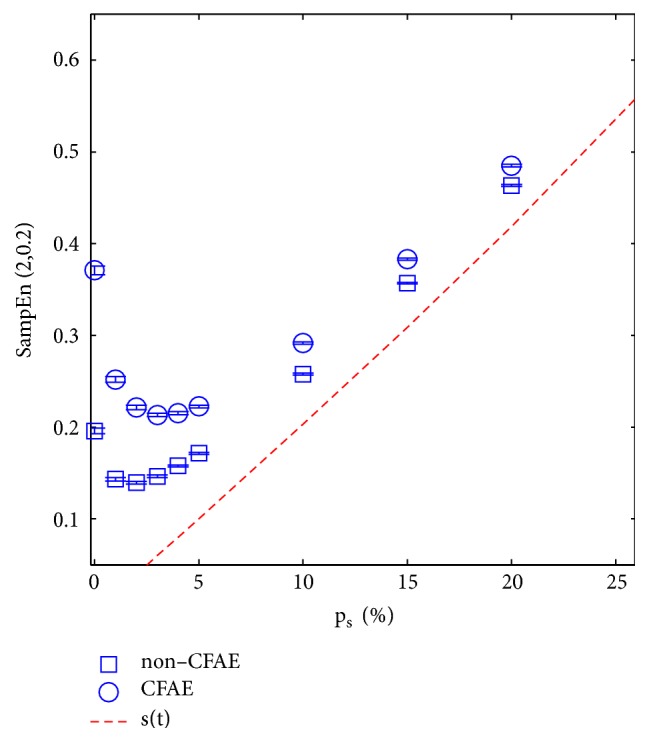
SampEn behaviour NC AEGM signals (box) and C AEGM signals (circle) when a spike train of probability *p*_*s*_ was superimposed to the signal. The red dashed line indicates the spike train entropy in terms of *p*_*s*_. Boxplots fall inside the boxes or circles, respectively, due to the low variance of the SampEn values.

**Figure 5:**
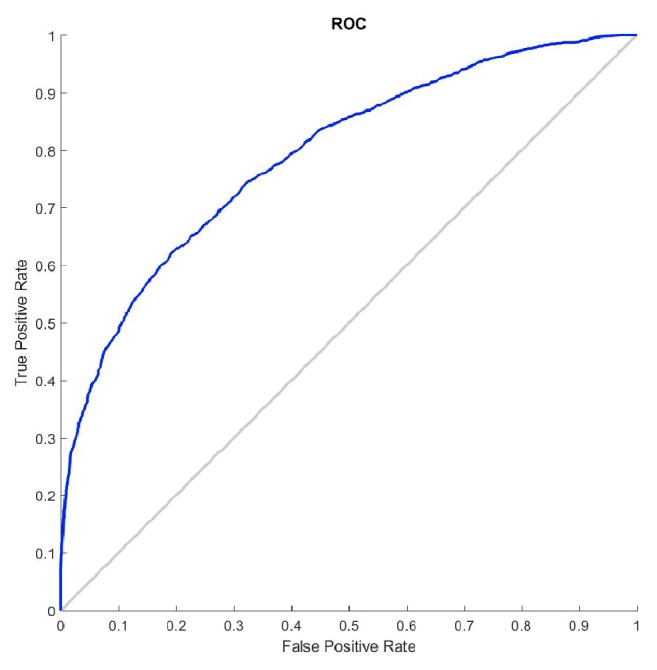
Influence of spikes on AEGM signals classification. ROC curve for *p*_*s*_ = 0.1.

Figure 4 depicts the influence that the inclusion of random spikes in AEGM signals had on SampEn values. When spikes were not present (*p*_*s*_ = 0) or found only in a small proportion (*p*_*s*_ < 0.1), both groups C and NC AEGM were statistically separated.  Figure 5 shows the corresponding ROC curve for the case *p*_*s*_ = 0.1. For larger *p*_*s*_, spikes masked the original AEGM signal entropy, and the ability to discern between AEGM fractionation was lost.

Table 1 shows, for different *p*_*s*_, the numerical results related to the characterisation of the C and NC AEGM signals entropy at different spike perturbation levels. The metric SampEn can be considered robust enough to provide a good interpretation of the AEGM complexity in the presence of spikes for *p*_*s*_ ≤ 0.10, with a correlation coefficient of *ρ*_*xy*_ > 0.8.

According to previous results, see Figure 4, the entropy of the spikes dominates the complexity of the artifacted signal for *p*_*s*_ above 0.10. The complexity of this signal exhibits the same behaviour as the regularity of the spike trains. Table 1 shows that, above this percentage, the measure should not be considered robust enough (*ρ*_*xy*_(15%) < 0.8) [26].

### 3.3. Sample Loss Influence on SampEn

#### 3.3.1. Distributed Random Sample Loss

**Figure 6:**
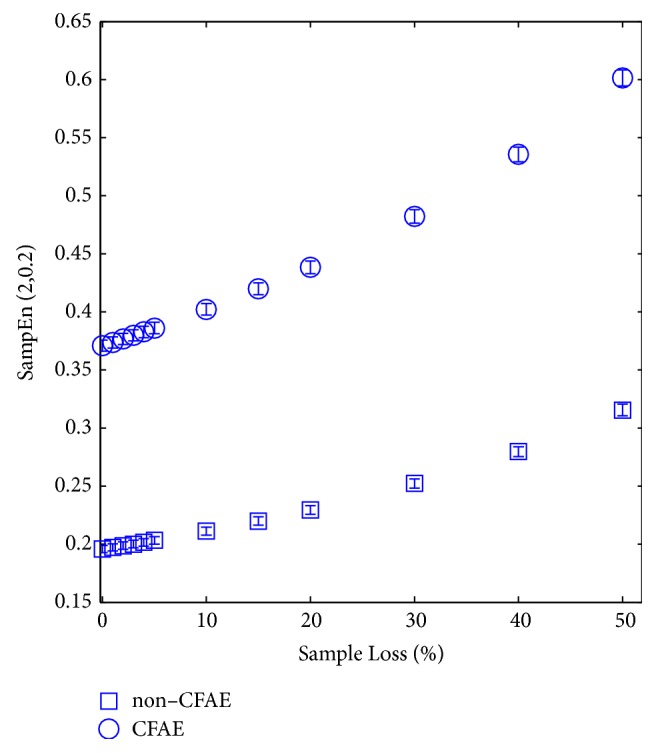
SampEn behaviour in terms of the percentage of random sample loss for NC (box) and C (circle) AEGM signals. Boxplots fall inside the boxes or circles respectively due to the narrow variance of the SampEn values.

**Figure 7:**
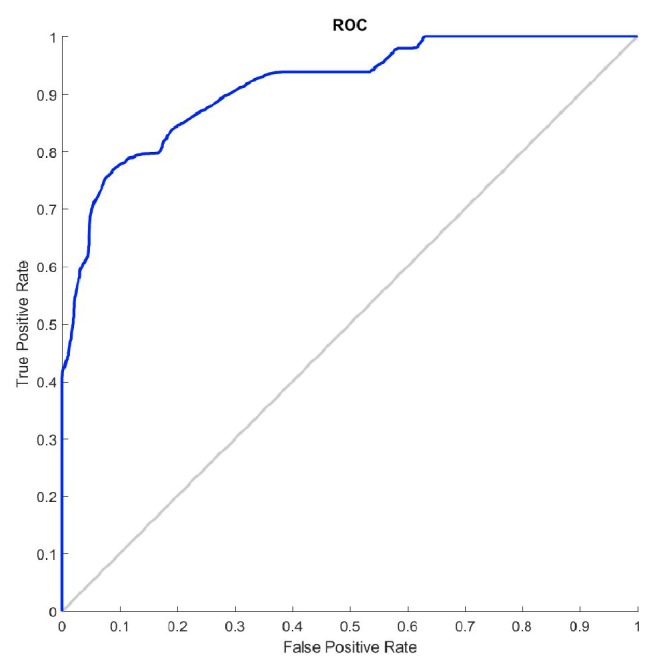
Influence of random sample loss on AEGM signals classification. ROC curve for *η* = 10%.

Figure 6 shows the behaviour of SampEn when AEGM undergo distributed random sample loss. It shows that AEGM complexity increases proportionally to the number of lost samples.  Figure 7 depicts the corresponding ROC curve for *η* = 10%. The relationship between SampEn and the sample loss ratio can be accurately modelled linearly, *f*(*η*) = 0.002*η* + 0.19 for NC records, and *f*(*η*) = 0.004*η* + 0.36 for the C AEGM records, with an adjustment of 0.981 and 0.985, respectively, and a standard error less than 1% in both cases.

Finally, in Table 2, a statistical characterisation of each class for some considered sample loss ratios is given. Mean values are different enough to obtain a significant segmentation probability (*p*-value< 0.001) and the measure is robust enough to characterise these signals, even though half of the signal was removed (*ρ*_*xy*_ > 0.80,  *p*-value< 0.001).

Table 2 shows, for the different *η* values, the numerical results related to the characterisation of the C and NC AEGM signals entropy at different distributed random loss levels. The metric SampEn can be considered robust enough to provide a good interpretation of the AEGM complexity, even with missing epochs for *η* ≤ 50%, at a correlation coefficient of *ρ*_*xy*_ > 0.9.

#### 3.3.2. Consecutive Random Sample Loss

**Figure 8:**
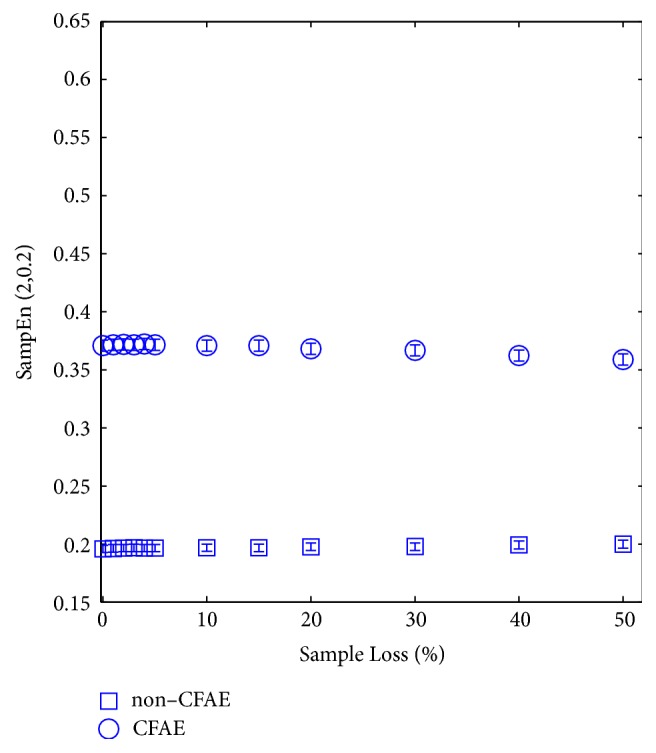
SampEn behaviour when consecutive sample loss occurs in the NC (square) and C (circle) AEGM signals. Boxplots fall inside the boxes or circles due to the narrow variance of the SampEn values.

**Figure 9:**
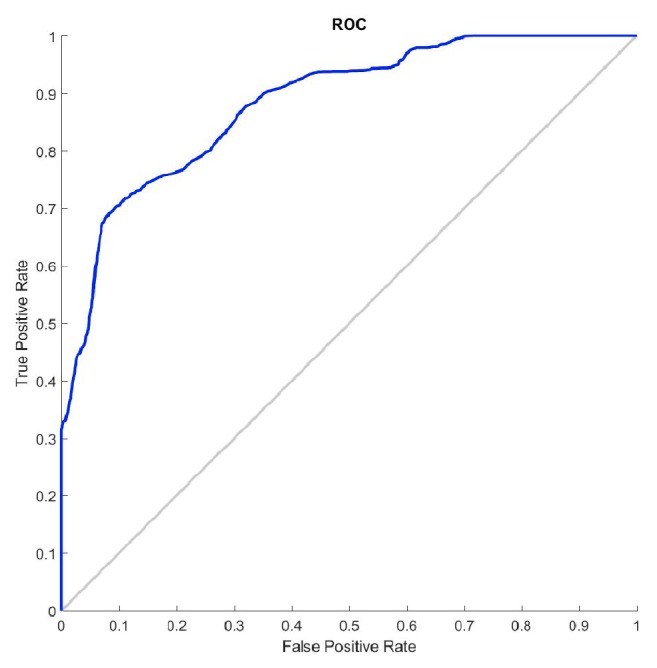
Influence of consecutive sample loss on AEGM signals classification. ROC curve for *η* = 10%.

Figure 8 shows the evolution of SampEn values for NC and C AEGM when consecutive sample loss takes place. Unlike distributed random sample loss (Figure 6), this time SampEn remains more or less constant for a wide range of percentages. A slow and small SampEn decrease beyond 15% of sample loss is found in C signals, which is not observed for the NC signals. SampEn can be characterised as constant within a given range with consecutive random sample loss.  Figure 9 depicts the corresponding ROC curve for *η* = 10%.

Table 3 provides the statistical characterisation of SampEn for some analysed percentages. Similar results to the distributed sample loss were found. Classes can be separated with statistical validity (*p*-value< 0.001). SampEn remained robust (*ρ*_*xy*_ > 0.9) and unchanged, even though samples were removed.

## 4. Discussion

All the experiments in this paper used a standard parameter configuration for SampEn, as suggested by [27] for ApEn. Other works have also used similar parameter configurations. In [28], the authors used *m* = 2 and *r* = 0.25 to compute SampEn complexity on paroxysmal AF. This work characterised both paroxysmal and persistent AF with no further consideration. In [29], the region inside 1 ≤ *m* ≤ 5 and 0.1 ≤ *r* ≤ 0.6 was considered appropriate for the same purpose. Therefore, it can be arguably reasonable to use the parameter configuration proposed herein.

In the baseline experiment, without external perturbations, SampEn was able to discern between classes. The results in Figure 3 show that the median values for C and NC clearly differ and can be statistically separated (*p*-value< 0.001). Thus SampEn is an appropriate measure to quantify the system complexity of AEGM signals, even with a short record length of 1.5s only. This length is a study limitation, and performance is arguably likely to improve with longer records, provided they are sufficiently stable.

The influence of spikes on the entropy of AEGM signals was characterised and quantified using synthetic spike trains added to the original signals. The results shown in Figure 4 and Table 1 account for SampEn performance under these conditions. For *p*_*s*_ > 0.10, it would be necessary to apply a signal processing technique to minimise the spike influence since its entropy supersedes that of the underlying record, and the measure loses its interpretability [26]. The results depicted in Figure 4 are similar to those obtained in [10] for the simulated ECG and RR signals. Firstly, SampEn abruptly drops to reach a minimum, from which it begins to increase. The drop is associated with an increase in the number of matches of length *m* + 1 because the randomness introduced by the spike tends to regularise the signal, but when spike probability increased, the number of matches of length *m* + 1 lowered. Thus complexity increased [19, 27]. In this case, SampEn did not measure the entropy of AEGM, but the entropy of the spike train.

Finally, the influence of distributed or contiguous sample loss was assessed. Previous works dealing with EEG signals have shown good performance for SampEn in this context [17]. The changes in performance observed for distributed sample loss are coherent with those presented in [17, 18, 20], where complexity increased due to a rise in randomness that removing samples introduced.

The expected behaviour in consecutive sample loss implied that complexity should be kept more or less constant as removing a segment of a signal implies removing approximately the same number of matches of length *m* and of length *m* + 1, so the ratio in (4) should be similar to the case before removal. However, this might render the record too short for an accurate SampEn estimation and, therefore, this prior assumption has to be validated. Figure 8 and Table 3 confirmed this expected behaviour, but with complexity of C signals slightly decreasing for the sample loss ratios higher than 15%, which is the same ratio as in [20], and with the same SampEn parameters, despite dealing with AEGM signals instead. In [20], signal epochs were removed from heart rate signals, and heart rate variability was analysed. This could be due to the bias that both ApEn and SampEn showed for short signal records [21] but could also be associated with the remaining correlation between the vectors that *d*_*ij*_ compared [30].

## 5. Conclusions

This study addressed the regularity characterisation of the AEGM signals recorded in RFA procedures of AF and their associated SampEn. It assessed the metric capability to distinguish between C and NC AEGM and provided insight into the influence of spikes or sample loss.

From the results, we conclude that

- SampEn is an appropriate regularity measure for AEGM signals as it enables the robust segmentation between C and NC regions. Hence this measure can be used in future clinical studies to prove that some RFA regions can be located by SampEn much more quickly and accurately. Furthermore, the method can be used in a real-time application as it provides reliable results, even on short records (1,500 ms) and exhibits a lower computational cost than other regularity measures such as ApEn or DFA;
- when analysing the AEGM signals corrupted with spikes, if their frequency of occurrence is relatively low (10%), SampEn can be used without having to apply any prior processing as SampEn proved able to separate between classes NC and C. If more spikes are present, it is advisable to filter spikes out as much as possible because their influence may blur class separability;
- SampEn is very robust to any type of sample loss and is able to separate between classes, even if the 50% of the samples are lost.

## Figures and Tables

**Table 1:** SampEn statistical characteristics for both classes NC AEGM and C AEGM when a spike train of probability *p*_*s*_ is added. For each *p*_*s*_, the statistical probability related to the separability between classes (*p*-value), the confidence intervals (CI) at 95%  (*μ* ± 2*σ*), and the cross correlation coefficient *ρ*_*xy*_ between the SampEn values of the initial signal and the signal corrupted with spikes are given.

*p*_*s*_	0.01	0.05	0.10	0.15
*p*-value	0.01	0.001	0.001	0.001
CI NC	0.196 ± 0.003	0.158 ± 0.001	0.171 ± 0.001	0.258 ± 0.001
CI C	0.371 ± 0.005	0.215 ± 0.002	0.223 ± 0.002	0.291 ± 0.001
*ρ*_*xy*_	1	0.887	0.863	0.705

**Table 2:** SampEn statistical characteristics for both classes NC AEGM and C AEGM when distributed random sample loss occurs. For each *η*, the statistical probability related to the separability between classes (*p*-value), the confidence intervals (CI) at 95%  (*μ* ± 2*σ*), and the cross correlation coefficient *ρ*_*xy*_ between the SampEn values of the initial signal and the signal with sample loss are given.

*η*(%)	0	10	30	50
*p*-value	0.001	0.001	0.001	0.001
CI NC	0.196 ± 0.003	0.211 ± 0.003	0.252 ± 0.004	0.315 ± 0.005
CI C	0.371 ± 0.005	0.402 ± 0.005	0.482 ± 0.006	0.602 ± 0.007
*ρ*_*xy*_	1	0.999	0.996	0.987

**Table 3:** SampEn statistical characteristics for both classes NC AEGM and C AEGM when consecutive sample loss was applied. For each *η*, the statistical probability related to the separability between classes (*p*-value), the confidence intervals (CI) at 95%  (*μ* ± 2*σ*), and the cross correlation coefficient *ρ*_*xy*_ between the SampEn values of the initial signal and the signal with sample loss are given.

*η*(%)	0	10	30	50
*p*-value	0.001	0.001	0.001	0.001
CI NC	0.196 ± 0.003	0.197 ± 0.003	0.198 ± 0.003	0.200 ± 0.003
CI C	0.371 ± 0.005	0.371 ± 0.005	0.367 ± 0.005	0.359 ± 0.005
*ρ*_*xy*_	1(0.001)	0.996(0.001)	0.981(0.001)	0.953(0.001)

## Data Availability

Data can not be publicly available due to patient confidentiality.
